# The durability of natural infection and vaccine-induced immunity against future infection by SARS-CoV-2

**DOI:** 10.1073/pnas.2204336119

**Published:** 2022-07-15

**Authors:** Jeffrey P. Townsend, Hayley B. Hassler, Pratha Sah, Alison P. Galvani, Alex Dornburg

**Affiliations:** ^a^Department of Biostatistics, Yale School of Public Health, New Haven, CT 06510;; ^b^Department of Ecology and Evolutionary Biology, Yale University, New Haven, CT 06525;; ^c^Program in Computational Biology and Bioinformatics, Yale University, New Haven, CT 06511;; ^d^Program in Microbiology, Yale University, New Haven, CT 06511;; ^e^Center for Infectious Disease Modeling and Analysis, Yale School of Public Health, New Haven, CT 06525;; ^f^Department of Epidemiology of Microbial Diseases, Yale School of Public Health, New Haven, CT 06520;; ^g^Department of Bioinformatics and Genomics, University of North Carolina, Charlotte, NC 28223

**Keywords:** SARS-CoV-2, COVID-19, vaccine, immunity, antibody

## Abstract

The durability of vaccine-mediated immunity to SARS-CoV-2, the durations to breakthrough infection, and the optimal timings of booster vaccination are crucial knowledge for pandemic response. Here, we applied comparative evolutionary analyses to estimate the durability of immunity and the likelihood of breakthrough infections over time following vaccination by BNT162b2 (Pfizer-BioNTech), mRNA-1273 (Moderna), ChAdOx1 (Oxford-AstraZeneca), and Ad26.COV2.S (Johnson & Johnson/Janssen). We evaluated anti-Spike (S) immunoglobulin G (IgG) antibody levels elicited by each vaccine relative to natural infection. We estimated typical trajectories of waning and corresponding infection probabilities, providing the distribution of times to breakthrough infection for each vaccine under endemic conditions. Peak antibody levels elicited by messenger RNA (mRNA) vaccines mRNA-1273 and BNT1262b2 exceeded that of natural infection and are expected to typically yield more durable protection against breakthrough infections (median 29.6 mo; 5 to 95% quantiles 10.9 mo to 7.9 y) than natural infection (median 21.5 mo; 5 to 95% quantiles 3.5 mo to 7.1 y). Relative to mRNA-1273 and BNT1262b2, viral vector vaccines ChAdOx1 and Ad26.COV2.S exhibit similar peak anti-S IgG antibody responses to that from natural infection and are projected to yield lower, shorter-term protection against breakthrough infection (median 22.4 mo and 5 to 95% quantiles 4.3 mo to 7.2 y; and median 20.5 mo and 5 to 95% quantiles 2.6 mo to 7.0 y; respectively). These results leverage the tools from evolutionary biology to provide a quantitative basis for otherwise unknown parameters that are fundamental to public health policy decision-making.

## Significance

The durability of natural and vaccine-mediated immunity to future SARS-CoV-2 infection and the necessity of booster vaccination are crucial knowledge for pandemic response, yet these factors remain poorly understood. Here, we use comparative evolutionary approaches to estimate the durability of immunity and the likelihood of future infections over time following vaccination by messenger RNA (mRNA) and viral vector COVID-19 vaccines. These findings provide quantitative evidence supporting booster vaccination as a crucial approach toward the curtailment of breakthrough infections and reinfections.

The unprecedented development of efficacious vaccines against SARS-CoV-2 has represented a triumph in the global effort to control the ongoing COVID-19 pandemic. Vaccines have been shown to provide short-term protection from major adverse health outcomes of hospitalization and death (1–4). However, protection against breakthrough infection wanes (5), and breakthroughs have been extensively documented (6, 7). In response, the Food and Drug Administration advisory committee has recommended a booster of the Pfizer-BioNTech and Moderna vaccines at least 5 mo after completion of the primary series to people ≥12 and ≥18 y of age, respectively (8). A booster dose of the Johnson & Johnson/Janssen vaccine has been authorized on a faster timescale—as early as 2 mo after the single dose to individuals 18 y of age and older (8). Nevertheless, the optimal timing of boosting remains challenging to assess. Consequently, rigorous prediction of the durability of immunity conferred by vaccination against the SARS-CoV-2 virus is essential to personal and public health decision-making, having major implications regarding policy decisions about COVID-19 vaccination around the world (9, 10).

Short-term longitudinal studies of SARS-CoV-2-neutralizing antibodies in vaccinated individuals (11–13) can provide information crucial to our understanding of the durability of vaccine-mediated immunity. Peak antibody responses following vaccination versus natural responses have also been quantified (14), facilitating analytical comparison of initial immune responses. For endemic viruses, longitudinal data on reinfection can provide reinfection probabilities associated with antibody level. However, longitudinal data on SARS-CoV-2 reinfection are not available during the short term associated with pandemic spread. Nevertheless, longitudinal reinfection data for a diversity of coronaviruses have been collected (15–20). SARS-CoV-2 reinfection probabilities have been obtained from them by phylogenetic analysis, using continuous ancestral and descendent state estimation (21). These estimates, produced before reinfection was commonplace, proved accurate (predicting an 18% probability of reinfection at ∼270 d [ref. 21] that was validated by a subsequent empirical finding of 18% reinfection by 275 to 300 d after primary infection [ref. 22] and, likewise, predicting a 34% probability of reinfection at ∼450 d after primary infection [ref. 21] that was validated by a subsequent empirical finding of 34% breakthrough infection 420 to 480 d after primary vaccination [ref. 23]). Similar analyses pairing antibody response and rates of waning for each vaccine with infection probabilities can enable quantification of the durability of vaccine-mediated immunity against breakthrough infections. The aim of this study is to leverage data on antibody response to each vaccine and corresponding probabilities of infection to estimate the durability of vaccine-mediated immunity against breakthrough SARS-CoV-2 infection for four well-studied vaccines: mRNA-1273, BNT162b2, ChAdOx1, and Ad26.COV2.S.

## Methods

### Study Design.

We conducted analyses of antibody waning and breakthrough infection probabilities for two messenger RNA (mRNA) vaccines, BNT162b2 and mRNA-1273, and two viral vector vaccines, ChAdOx1 and Ad26.COV2.S. We applied a comparative evolutionary framework for inference of infection probability associated with antibody level after natural infection to the typical antibody response for these four vaccines relative to natural infection. We projected antibody waning profiles for each vaccine and antibody-associated infection probabilities to estimate probabilities of breakthrough infection through time that inform schedules of booster vaccination.

### Data Acquisition.

#### Phylogenetic tree topologies.

Phylogenetic relationships of SARS-CoV-2 and the endemic human-infecting coronaviruses were based on data from 58 *Alphacoronavirus*, 105 *Betacoronavirus*, 11 *Deltacoronavirus*, and three *Gammacoronavirus* lineages (21). We analyzed the concatenated alignment of the *S*, *M*, and ORF1b genes to reconstruct maximum-likelihood molecular phylogenies using IQ-TREE v2.0.6 (24) and RAxML v7.2.8 (25), with 1,000 nonparametric bootstrap replicates to assess node support. For each analysis, we specified a general time-reversible model of nucleotide substitution incorporating discretized gamma-distributed rate variation across sites and a proportion of invariable sites (GTR + I + Γ_4_). Topologies were time calibrated using least-squares dating (26) in IQ-TREE v2.0.6 (24), RelTime (27) in MEGA X v10.1.9 (28), and TreeTime v0.7.6, enabling us to assess consistency across divergence times that were scaled proportionally to the most recent common ancestor. We also repeated these phylogenetic analyses using nonrecombining blocks of sequence (29) that were realigned and analyzed using the methods identified above. Resulting topologies were robust to alternative phylogenetic likelihood search algorithms (24, 25), to alternative divergence time estimation approaches (24, 27, 28, 30 ), and to a potential history of recombination (29). Finally, an IQ-TREE v2.0.6 (24) maximum-likelihood molecular phylogeny and chronogram were inferred with the addition of four SARS-CoV-2 strains (GenBank accessions: OL986696.1, MZ286753.1, MW617734.1, MW422256.1) that are representative of the alpha, beta, delta, and omicron variants of concern. These analyses enabled us to quantify the evolutionary distance between these SARS-CoV-2 strains and their common ancestor compared to the evolutionary distance between that common ancestor and its common ancestor with other zoonotic and endemic coronavirus lineages.

#### Waning antibody data.

To obtain data that would provide relative peak antibody levels comparing BNT162b2 to mRNA-1273, ChAdOx1, Ad26.COV2.S, or natural infection that occurred at a known time relative to antibody measurement, we conducted literature searches using the PubMed and Google Scholar databases. Searches were conducted between 1 July 2021 and 31 January 2022 and used the names of each vaccine as search terms in combination with “SARS-CoV-2,” “antibodies,” “antibody response,” “Enzyme-Linked ImmunoSorbent Assay (ELISA),” “IgG,” (immunoglobulin G) “longitudinal,” “optical density,” “naïve,” “seropositive,” “natural infection,” or “convalescent.” There were no language restrictions imposed. Studies were included when they reported ELISA anti-Spike (S), anti-S1, or anti-Recombination Binding Domain (anti-RBD) data that covered the peak antibody response for naive individuals vaccinated with either mRNA vaccine compared to those vaccinated with either viral vector vaccine.

This dataset was then combined with a dataset assembled by Townsend et al. (21) on waning antibody levels following natural infection by SARS-CoV-2 and its closest human-infecting relatives (Dataset S1). To supplement the natural infection data gathered by Townsend et al. (21) with data that have recently become available, we conducted a literature search to identify additional studies of SARS-CoV-2 antibody waning following natural infection (Dataset S1). Searches were conducted on PubMed and Google Scholar databases between July 2021 and August 2021 with no language restriction imposed. “SARS-CoV-2,” in combination with “antibodies,” “antibody response,” “coronavirus,” “ELISA,” “IgG,” “immunity,” “immune response,” “longitudinal monitoring,” “optical density,” “Euroimmun,” “S protein,” “Spike protein,” “reinfection,” “serological,” and “titer” were used as search terms. Natural infection studies that used a consistent antibody type (Euroimmun S1) and provided longitudinal sampling were selected for inclusion, thereby ensuring that our comparative phylogenetic analyses were conducted on a common scale of immunological measurement (31). To obtain data for antibody-waning profiles following vaccination that are an alternative to the antibody-waning profile following natural infection, we conducted an additional literature search between 1 September 2021 and 31 January 2022 using vaccine names as search terms in combination with the longitudinal waning terms previously mentioned.

#### Waning antibody profiles and baselines.

We constructed profiles of SARS-CoV-2 anti-S1 IgG antibody waning through time as in Townsend et al. (21), except that the putatively cross-reactive data points from Edridge et al. (16) were excluded. We first extracted postpeak infection antibody levels for other human-infecting coronaviruses (Severe Acute Respiratory Syndrome Corona Virus (SARS-CoV-1), Middle-Eastern Respiratory Syndrome Corona Virus (MERS-CoV), Human Corona Virus (HCoV-OC43), HCoV-NL63, HCoV-229E) and SARS-CoV-2. These optical density (OD) values were normalized such that the typical postinfection peak antibody level in response to natural infection was quantified at 1.0 for each virus. Mathematica v12.0.0.6206964 was used to calculate a typical antibody waning profile, with baseline anti-Spike IgG values for SARS-CoV-2, SARS-CoV-1, and MERS-CoV estimated using continuous phylogenetic ancestral states analysis (32) applied toward descendent states via Rphylopars v0.2.12 (33). This approach estimates unobserved trait values for a taxon or taxa using a Brownian motion model of trait evolution along a phylogenetic tree and accounts for evolutionary covariance between species-specific parameters of traits (34)—in this case, parameters describing antibody waning rate λ and the probability of infection given antibody level in its descendent-state estimation.

We projected the time course for each typical antibody waning profile beyond the extant dataset to the duration of the longest full typical antibody waning profile inferred (HCoV-229E, 4,393 d postpeak infection). This augmented exponential projection of antibody waning was associated with a probability of infection using linear logistic regression of daily probability of infection against antibody level based on data from Edridge et al. (16). This analysis yields a probability of infection given by ${(1+e^{-\left(a+bg\right)})}^{-1}$ with parameters *a* (intercept) and *b* (slope), dependent on *g*, the peak-normalized antibody level (21).

Using Rphylopars v0.2.12, we estimated the *a* and *b* parameters for SARS-CoV-2, SARS-CoV-1, and MERS-CoV specifying as coevolving and correlated traits our quantifications of λ and their phylogenetically informed baseline antibody levels (21). In Mathematica, we composed a single antibody waning time course for each virus by following the typical antibody waning time course with projected antibody waning for each virus. Using these antibody waning time courses and the logistic infection functions inferred for each virus, we calculated the probabilities of infection on each day and the quantiles that correspond to the times by which 5, 50, and 95% of individuals would be expected to become reinfected under endemic conditions. Comprehensive custom Mathematica notebooks illustrating our approach and used to perform the analyses are available (35).

To connect these results on the durability of immunity against natural reinfection to durability of immunity against breakthrough infection, we quantified the ratio of typical peak anti-RBD IgG antibody levels associated with vaccination by BNT162b2 to peak anti-RBD IgG antibody levels associated with natural infection. We then projected waning beginning at this ratio (which is relative to natural infection normalized at 1.0). We assumed a rate of antibody waning above 1.0 that is consistent with that observed at 1.0. Phylogenetic ancestral and descendent analyses via Rphylopars (33) were repeated to assess the impact of method of phylogenetic inference on our phylogenetic trait estimation of the baseline antibody level ω and the linear logistic infection function parameters *a* and *b*. We compared results for linear logistic infection function parameters *a* and *b* and the baseline antibody level ω that were conditioned on the relative phylogenetic chronogram estimated in IQ-TREE to 1) the molecular phylogenies from IQ-TREE and RAxML analyses; 2) the relative phylogenetic chronograms estimated using RelTime and TreeTime; and 3) those phylogenies produced using the nonrecombinant alignment. These analyses were replicated to quantify durabilities of immunity against future infection specifying SARS-CoV-2 sequences representative of the alpha, beta, delta, and omicron strains in place of the original Wuhan strain of SARS-CoV-2.

To assess the impact of using alternate sources of anti-Spike IgG antibody data on our analyses, we replicated all of the above methodologies, performing five additional sets of evolutionary and statistical analyses, designated 2 through 6. For analyses 2 and 3, we substituted two alternate SARS-CoV-2 anti-S1 IgG OD longitudinal datasets (36, 37) and performed an analysis that was otherwise identical to analysis 1. For analyses 4, 5, and 6, we repeated analyses 1 through 3, substituting an alternate anti-S IgG OD dataset for MERS-CoV (18). To assess the choice of phylogenetic inference method, we repeated analyses 1 through 6, performed using the IQ-TREE phylogenetic chronogram, using the 13 additional phylogenies described previously: 9 additional phylogenetic chronograms and 4 molecular phylogenies. In total, these analyses resulted in 84 postpeak-median-time-to-reinfection estimates for SARS-CoV-2 and 336 postpeak-median-time-to-breakthrough-infection estimates (84 estimates for each vaccine).

To assess the impact of using alternate sources of vaccine waning antibody data on our analyses, we performed an additional analysis substituting the estimates of antibody waning following vaccination with estimates obtained by sample-size-weighted averaging across studies for vaccines with sufficient aggregate sample sizes. This average-weighted estimate was determined by first estimating the antibody waning for all available waning antibody data. Weighting each estimate by its sample size, the slopes within intervals of a width 5% of the peak antibody response after vaccination were averaged across studies.

To quantify an advisable timing of administering a booster to fully vaccinated individuals, we added the average number of days from the date of vaccination until the peak antibody response for each vaccine to the number of days from that peak until the accumulation of 5% probability of breakthrough infection under endemic conditions.

## Results

Complementing data comparing peak antibody levels of the BNT162b2 mRNA vaccine to that of natural infection (14), our literature search yielded nine additional studies that provided a common temporal and assay basis for comparative analysis. These studies comprise a total of 14 comparisons of anti-RBD, anti-S1, or anti-S IgG antibodies following vaccination between BNT162b2 and three other vaccines (Table 1): 6 comparisons between BNT162b2 and mRNA-1273 (38–43), 4 comparisons between BNT162b2 and ChAdOx1 (38, 39, 44, 45), and 4 comparisons between BNT162b2 and Ad26.COV2.S (40, 42, 43). Data from Horndler (38) regarding Ad26.COV2.S were excluded, as the duration of antibody response reported for the vaccine did not include the peak. Combined, these 10 studies provided anti-RBD, anti-S1, or anti-S IgG antibody data on the following:

**Table 1.** Peak antibody levels subsequent to vaccination and natural infection

Stimulus	Subjects	IgG antibody	Day*	Peak^†,^^‡^	Study
mRNA-1273	10	anti-S1	35	1.59125597	(39)
mRNA-1273	52	anti-S	28	1.421908903	(47)
mRNA-1273	8	anti-RBD	14	1.421860374	(46)
mRNA-1273	29	anti-S	14	1.481656668	(39)
mRNA-1273	40	anti-RBD	28	1.534648493	(42)
mRNA-1273	199	anti-S	28	1.551304142	(43)
	*Mean* (*across studies*)		24.5	1.500439092	*—*
BNT162b2	21	anti-S1	21	—^†^	(39)
BNT162b2	119	anti-S	28	—^†^	(47)
BNT162b2	109	anti-S	28	—^†^	(44)
BNT162b2	3	anti-S	14	—^†^	(48)
BNT162b2	29	anti-S	28	—^†^	(40)
BNT162b2	54	anti-S	21	—^†^	(45)
BNT162b2	21	anti-RBD	14	—^†^	(46)
BNT162b2	50	anti-RBD	28	—^†^	(42)
BNT162b2	100	anti-S	28	—^†^	(43)
BNT162b2	3808	anti-RBD	30	—^†^	(49)
BNT162b2	231	anti-RBD	7	—^†^	(50)
BNT162b2	309	anti-S	14	—^†^	(51)
BNT162b2	379^¶^	anti-S	0–29	—^†^	(52)
BNT162b2	1256	anti-S	37	1.500037185	(14)
	*Mean* (*across studies*)		22.7	1.500037185	—
Natural infection	1797	anti-S1	34	1.00000^‡^	(53)
Natural infection	264	anti-S1	28	1.00000^‡^	(36)
Natural infection	145	anti-S1	56	1.00000^‡^	(37)
Natural infection	272	anti-S	40	1.00000^‡^	(14)
	*Mean* (*across studies*)		39.5	1.00000^‡^	—
ChAdOx1	29	anti-S1	35	0.886550776	(39)
ChAdOx1	39	anti-S	28	1.125027225	(47)
ChAdOx1	104	anti-S	28	1.136666043	(44)
ChAdOx1	77	anti-S	14 to 21	1.250836015	(45)
	*Mean* (*across studies*)		25.2	1.099770015	—
Ad26.COV2.S	15	anti-S	56	0.974409364	(40)
Ad26.COV2.S	75	anti-S	56	0.953157648	(43)
Ad26.COV2.S	13	anti-RBD	56	0.830522868	(42)
	*Mean* (*across studies*)^§^		56	0.919363294	*—*

^*^Days after final vaccine dose (second dose for mRNA-1273, BNT162b2, and ChAdOx1 or singular dose for Ad26.COV2.S) or average days post-symptom onset for natural infection.

^†^Reference values versus natural infection, against which all other vaccines were compared.

^‡^Value for natural infection assigned to equal 1.

^§^Antibody levels reported for Ad26.COV2.S by Horndler and colleagues (38) were not included in our quantification of the peak antibody level relative to natural infection for Ad26.COV2.S because the sampling of subjects was timed such that measurements did not necessarily represent the peak of response.

^¶^Average number of participants across sampling duration from Israel and colleagues (53).

1) 272 individuals infected with SARS-CoV-2 sampled at an average of 40 d after symptom onset and 1,256 individuals sampled 37 d after BNT162b2 vaccination (14)
2) 21 individuals sampled 2 wk after BNT162b2 vaccination and 8 individuals sampled 2 wk after mRNA-1273 vaccination (46)
3) 50 individuals sampled 4 wk after BNT162b2 vaccination, 40 individuals sampled 4 wk after mRNA-1273 vaccination, and 13 individuals sampled 56 d after Ad26.CoV2.S vaccination (42)
4) 100 individuals sampled 4 wk after BNT162b2 vaccination, 199 individuals sampled 4 wk after mRNA-1273 vaccination, and 75 individuals sampled 56 d after Ad26.CoV2.S vaccination (43)
5) 119 individuals sampled 4 wk after BNT162b2 vaccination, 52 individuals sampled 4 wk after mRNA-1273 vaccination, and 39 individuals sampled 4 wk after ChAdOx1 vaccination (38)
6) 21 individuals sampled 3 wk after BNT126b2 vaccination, 10 individuals sampled 5 wk after mRNA-1273 vaccination, and 29 individuals sampled 5 wk after ChAdOx1 vaccination (39)
7) 29 individuals sampled 4 wk after BNT162b2 vaccination, 32 individuals sampled 4 wk after mRNA-1273 vaccination, and 15 individuals sampled 8 wk after Ad26.COV2.S vaccination (40)
8) 3 individuals sampled 2 wk after BNT162b2 vaccination and 29 individuals sampled 2 wk after mRNA-1273 vaccination (41)
9) 54 individuals sampled 2 to 3 wk after BNT162b2 vaccination and 77 individuals sampled 2 to 3 wk after ChAdOx1 vaccination (45)
10) 109 individuals sampled 4 wk after BNT162b2 vaccination and 104 individuals sampled 4 wk after ChAdOx1 vaccination (44)

For all nine studies, time after vaccination refers to the number of weeks after the second dose of BNT162b2, mRNA-1273, and ChAdOx1 or after the singular dose of Ad26.COV2.S. By quantifying the peak antibody levels for each vaccine relative to peak antibody levels following natural infection, we found that mean antibody response to the mRNA vaccines mRNA-1273 and BNT162b2 exceeded the mean antibody response to natural infection. In contrast, the mean antibody response to viral vector vaccines ChAdOx1 and Ad26.COV2.S was lower than the mean antibody response to these mRNA vaccines and similar to that of natural infection (Table 1).

Our literature search regarding antibody data subsequent to natural infection identified two additional studies (36, 37) that met the criterion of having sufficient ELISA optical density data on anti-S1 IgG antibody levels beyond the anti-S1 IgG antibody level dataset provided by Townsend et al. (21). In combination, these studies yielded six comparative datasets that provided insight into the durability of immunity as well as into the robustness of our findings to data selection (Dataset S1). Dataset S1 comprised anti-S1 data from a population sample of 1,797 individuals extending over 125 d after diagnosis of infection by SARS-CoV-2 (53); 9 individuals (5 males and 4 females; ages 27 to 54 y) infected by MERS-CoV with symptoms ranging from asymptomatic to severe, monitored up to 18 mo (15); and putative endemic coronavirus anti-S1 IgG antibody waning data from our linear model relating anti-N and anti-S1 IgG that included 10 adult males aged 27 to 75 y who were assayed for antibody response to infection by HCoV-OC43, HCoV-NL63, and HCoV-229E over 28 y spanning two periods: 1984 to 1997 and 2003 to 2020 (16). Datasets S2 and S3 included alternate SARS-CoV-2 data from two sources: (Dataset S2) 264 individuals over 28 wk whose positive status was validated by two or more assays in addition to the Euroimmun anti-S1 assay (36) and (Dataset S3) 145 seropositive health care workers who experienced infection over the course of 21 wk (37). Datasets S4–S6 were replicates of Datasets S1–S3 with the supplementation of MERS-CoV data from 11 individuals (5 with severe disease and 6 with mild disease) monitored over 1 y after symptom onset (18).

**Fig. 1.**
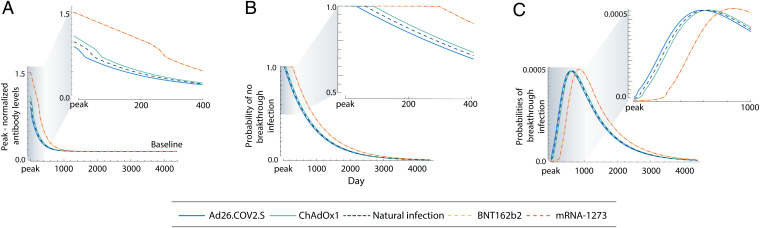
Peak-normalized anti-S1 IgG antibody levels; probabilities of no breakthrough infection given antibody level; and probabilities of natural reinfection or breakthrough infection for Ad26.COV2.S, ChAdOx1, BNT162b2, and mRNA-1273 vaccinations against SARS-CoV-2 infection over 4,000 d postpeak response. (*A*) Peak-normalized anti-S1 IgG antibody levels for vaccination with Ad26.COV2.S (blue), vaccination with ChAdOx1 (green), natural infection (navy, dashed), vaccination with BNT162b2 (yellow, dashed), and vaccination with mRNA-1273 (orange, dashed) against SARS-CoV-2 infection over 4,000 d postpeak response and (*Inset*) over the first 400 d (just over 1 y) postpeak response. (*B*) Probability of no natural reinfection or no breakthrough infection for Ad26.COV2.S, ChAdOx1, BNT162b2, and mRNA-1273 vaccinations against SARS-CoV-2 infection over 4,000 d postpeak response and (*Inset*) over the first 400 d postpeak response. (*C*) Probabilities of natural reinfection or breakthrough infection for Ad26.COV2.S, ChAdOx1, BNT162b2, and mRNA-1273 vaccinations against SARS-CoV-2 infection over 4,000 d postpeak response and (*Inset*) over the first 1,000 d postpeak response.

Our literature search conducted to substitute natural infection antibody waning profiles with vaccine profiles yielded four studies with sufficient sampling breadth (*n* = 231 to 3,808): 1) anti-RBD data from 3,808 individuals sampled over 165 d after vaccination with BNT162b2 (49), 2) anti-RBD data from 231 individuals sampled over 159 d after vaccination with BNT162b2 (50), 3) anti-S data from 309 individuals sampled over 270 d after BNT126b2 vaccination (51), and 4) anti-S data from a time-variable sample of 170 to 556 individuals over 180 d after BNT162b2 vaccination (52). These vaccine antibody waning profiles were subsequently averaged, weighting each study by its sample size (Dataset S2). Projection of the waning antibody levels postpeak in response to natural infection by SARS-CoV-2 exhibited consistent estimates of half-life to baseline, ranging from 36 to 156 d between datasets. These results were consistent regardless of the method of phylogenetic inference or whether a chronogram or a molecular evolutionary tree was used (Dataset S3). Results of the ancestral- and descendent-states analysis of the logistic regression parameters for the time-dependent probabilities of reinfection identified the relationships between the antibody waning profile (Fig. 1*A*) and the probabilities of reinfection or breakthrough infection under endemic conditions (Fig. 1*B*). Breakthrough infections in those vaccinated by either mRNA-1273 or BNT162b2 were predicted to typically occur after a longer period than natural reinfections. Vaccination by either Ad26.COV2.S or ChAdOx1, in contrast, provided probabilities of remaining infection free through time that were similar to natural infection (Fig. 1*B*). This difference in the probabilities of remaining free of infection over time among mRNA vaccines, compared to natural infection or the other two vaccines, corresponds to substantial differences in daily risks of breakthrough infections over time (Fig. 1*C*).

**Fig. 2.**
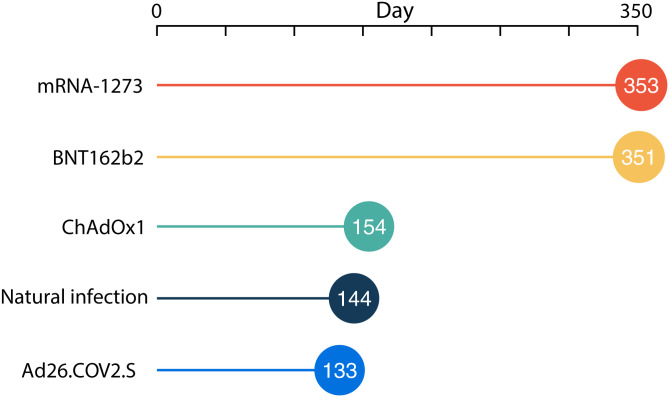
Mean time to 5% cumulative risk of natural reinfection or breakthrough infection under endemic conditions for mRNA-1273, BNT162b2, ChAdOx1, and Ad26.COV2.S vaccinations against SARS-CoV-2.

Consistent with Townsend et al. (21), the median time to reinfection postpeak antibody response for SARS-CoV-2 following natural infection is estimated to be 19.2 to 32.3 mo, depending on the composition of the dataset. Results varying by all permutations of SARS datasets, MERS dataset, and phylogeny are reported in Dataset S4 and *SI Appendix*, Fig. S1. Our primary analysis provides 5 to 95% quantiles for reinfection of 3.5 mo to 7.1 y postpeak antibody response. For both mRNA vaccines, the median time until vaccinated breakthrough infection exceeded the median time of unvaccinated natural reinfection. Alternate compositions of the antibody waning datasets produce estimates ranging from 22.0 to 33.6 mo for both mRNA-1273 and BNT162b2 (Fig. 1*B* and Dataset S4 and *SI Appendix*, Fig. S1). The 5 to 95% quantiles of breakthrough infection spanned a typically later and distinctly wider range for our primary analysis (10.9 mo to 7.9 y; Fig. 1*C*). Analyses of viral vector vaccines yielded median times to breakthrough infection for alternate compositions of 19.9 to 32.4 mo for ChAdOx1 and 18.7 to 32.1 mo for Ad26.COV2.S (5 to 95% quantiles of 4.3 mo to 7.2 y and 2.6 mo to 7.0 y, respectively), indicating a higher risk of breakthrough infection for these two vaccines. The 5 to 95% quantiles for SARS-CoV-2 natural reinfection and each vaccination type were very similar in three datasets composed from alternate sources (Datasets S5 and S6). There is negligible divergence between alternate SARS-CoV-2 pandemic virus strains relative to their divergence with common ancestors of distinct coronavirus lineages (*SI Appendix*, Fig. 2). Therefore, substitution of the original Wuhan strain of SARS-CoV-2 with alternate variants of concern (alpha, beta, delta, and omicron; Dataset S7) and inference on the basis of the consequent molecular evolutionary tree yielded no differences in median times to reinfection or breakthrough infection.

With no additional stimulation of the immune system, the mean time by which there is a 5% cumulative risk of reinfection under endemic conditions is 143.5 d postsymptom onset. (Fig. 2). This estimate is just over half of the estimated time until a cumulative 5% risk of breakthrough infection despite vaccination with mRNA1273 or BNT162b2 (352.5 and 350.7 d postvaccination, respectively). In contrast, a cumulative 5% risk of breakthrough infection accrues within 154.2 or 133.0 d postvaccination for ChAdOx1 and Ad26.COV2.S, respectively (Fig. 2).

## Discussion

Here, we have quantified the waning of antibodies, the probability of infection given antibody level under endemic conditions, and the distribution of likely times to reinfection or breakthrough infection following vaccination with mRNA-1273, BNT1262b2, ChAdOx1, or Ad26.COV2.S. Our analyses reveal a typical expectation of higher peak antibody levels following either of two full mRNA vaccinations relative to natural infection and either of two viral vector vaccinations. Antibodies from natural infection and vaccination are all projected to wane, associated with significant loss of protection and increasing probabilities of future infection. However, the predicted median time to breakthrough infection following mRNA vaccination (29.6 mo after vaccination with BNT162b2 or mRNA-1273) is longer than the median time to reinfection following natural infection (21.5 mo) and longer than the median time to breakthrough infections following viral vector vaccination (20.5 or 22.4 mo after vaccination with Ad26.COV2.S or ChAdOx1, respectively). These median times to breakthrough infection align with the relative efficacies of these vaccinations in their clinical trials (1–4).

These findings provide guidance on the timing of vaccination following natural infection to minimize the risk of reinfection and on the provision of booster doses to individuals who have been vaccinated with mRNA or viral vector vaccines to prevent breakthrough infections. To allow no more than a 5% probability of future infection as a consequence of waning immunity, vaccination of those whose only exposure was a natural infection should occur within 5 mo. To convey the same benefit, those fully vaccinated with either mRNA vaccine and no other exposure should receive a booster within a year, those fully vaccinated with Ad26.COV2.S should receive a booster by 4.5 mo, and those fully vaccinated with ChAdOx1 should receive a booster within 5 mo. These findings provide quantitative guidance for vaccination of unvaccinated but previously infected individuals, as well as for implementing booster vaccination programs. Policy makers with greater aversion to risk might consult our projections and set earlier goals for vaccination of those who have been naturally infected and for boosting of vaccination, whereas policy makers with lower aversion to risk might set later goals.

Studies have demonstrated that protection conferred by booster vaccination wanes (54–56). Currently, the data necessary to use this approach to evaluate the durability of immunity following booster vaccination are not available to expand our analyses. Timely booster vaccination could be presumed to lead to a greater antibody response than the initial vaccination in most individuals (57). Consequently, our approach would predict that third doses would result in a renewed immunity that endures for a period that is longer than the period of immunity conferred by the initial vaccination. However, the extension of this renewed durability beyond the period conferred by the initial vaccination would likely be fairly modest: The postpeak decline of antibody level is nearly exponential, which brings higher antibody levels relatively quickly into the same antibody-level domain as initial vaccination.

These timings of vaccination or boosters are appropriate to the context of a typical individual with typical antibody response to vaccination. However, immunocompromised individuals exhibit lower levels of antibody response to COVID-19 vaccination (58, 59); their immunity will likely wane to a point of significant risk at an earlier date postinfection or postvaccination. Consequently, accelerated boosting for such individuals with lower vaccine response is appropriate. A similar early waning of protection—in addition to higher risk of severe outcome—might be expected among the elderly or those with specific morbidities, necessitating earlier administration of booster doses to prevent future infections. Cohort-specific data on vaccination response would empower comparative analyses that provide increased resolution to the problem of when to administer boosters among groups with distinct health status.

Our results should be interpreted with consideration of unmodeled aspects of vaccine design, manufacturing, and antigen targeting. On the one hand, as a consequence of vaccine design and production, immunity following vaccination is “out of phase” with the evolution of its antigenic target. Vaccines typically go through several phases of clinical trials prior to deployment. However, during this time, viral antigens will continue to accrue new sequence variation that will gradually or abruptly shift them away from the target of the vaccine. Consequently, the immune response to a vaccine will, by necessity, target an earlier strain of the pathogen than would the immune response to a contemporaneous natural infection. This difference in strain targeting will mean that immune evasion by the virus will likely be more advanced against immunity conveyed by a vaccine than against immunity conveyed by natural infection. Indeed, neutralization efficacy of the ancestrally targeted SARS-CoV-2 vaccines against successive selected strains declined precipitously against the insurgent delta and omicron variants (57), and natural infection by a predominant strain has been shown to update vaccine-mediated immune targeting, increasing the antigenic breadth of neutralizing capacity (60).

On the other hand, vaccines can be designed to target conserved components of the exposed viral proteome that exhibit an especially slow rate of antigenic change. Host immune systems often target the most antigenic component of the exposed viral proteome, which, in turn, is often antigenically fast evolving. This evolutionary dynamic arises because viruses whose most antigenic components are especially fast evolving are most likely to be successful at reinfection and long-term persistence in a host population. However, immunity conveyed by vaccines that target conserved components is likely to last longer than immunity from natural infection. A longer durability of neutralization efficacy would slow the emergence of escape variants. Accordingly, efforts to develop vaccines and boosters that confer a greater durability of immunity by targeting antigenic genes or gene regions that are slow evolving represent an exciting prospect in mRNA vaccine design (61, 62).

Our study has several limitations. First, our study was limited by the availability of longitudinal data gathered on anti-S IgG antibody responses to SARS-CoV-2 infection or vaccination that could be compared via a consistent immunological assay across datasets. Antibody declines and infection probabilities used in our analyses are averaged among infected individuals and mask important sources of individual variation that include age, immune status, infection severity (for the naturally infected), vaccine response (for the vaccinated), cross-immunity, and other immunological factors such as T- and B-cell memory (63–65). Natural immunity and vaccine-mediated immunity may differ in major components of mechanistic protection, such as antibody neutralizing capacity over time (66). Our approach does not account for these factors or their interactions. However, there is a strongly supported statistical correlation between antibody levels and the risk of reinfection (21), and vaccines have been demonstrated to provide robust T- and B-cell responses (67, 68). Moreover, recent research has indicated that antibodies are the dominant component of the immune system providing protection against infection by SARS-CoV-2 (69), justifying their use in the context of quantifying the durability of immunity due to natural infection or vaccination.

Our continuous model of the declining probability of infection associated with lowering levels of antibodies appears inconsistent with observed discrete decreases in immunity to evolving variants of SARS-CoV-2 during this pandemic. However, all endemic coronaviruses, not only SARS-CoV-2, evolve escape variants to our immunological memory (70). Our models of endemic coronavirus probabilities of infection given antibody level not only reflect decreasing defense against infection over time as a consequence of decreasing antibody level, but also simultaneously incorporate average decreases in antibody efficaciousness against newly evolved variants in endemic coronaviruses (21). This synchroneity arises directly from our use of long-term longitudinal infection surveillance data to estimate the probabilities of infection given antibody level. Underlying that longitudinal data, there is also the continuous evolution of variants enabling novel infections to overcome immunological memory. Therefore, the challenge to vaccine manufacturers is to keep pace not only by boosting declining antibody levels, but also by vaccinating against emergent variants—just as we currently do with yearly influenza vaccinations (71).

Our probabilities of infection associated with antibody level are inferred from evolutionary expectations drawing on probabilities of infection quantified by longitudinal study of endemic—rather than pandemic—human-infecting coronaviruses. During a pandemic, populationwide immunological naiveté and diverse public health interventions exert strong opposing influences on disease incidence. Depending on the level of pandemic intervention, expectations of probabilities of infection can scale, fluctuate, and vary strikingly by location. With minimal intervention, incidence is likely higher than under endemic conditions due to immunological naiveté, providing more opportunities for infection. In this minimal-intervention case, our estimates are below the relevant probabilities of infection that should be expected to occur on an accelerated timescale. On the other hand, highly efficacious interventions such as lockdowns or sudden, widespread vaccination can greatly diminish incidence to levels below those observed under endemic conditions (72, 73). In these high-intervention scenarios, our estimates likely exceed the probabilities of future infection relative to probabilities estimated under endemic transmission. Low and high levels of public health intervention are occurring simultaneously across the globe (74). Therefore, all of our estimates should be interpreted cognizant of their context.

These first estimates of the durability of immunity following vaccination provide essential knowledge to policy decision-making that can curb transmission long term, mitigating morbidity and mortality consequent to SARS-CoV-2 infection. Our quantitative estimates will be improved as data on long-term immunological responses to SARS-CoV-2 infection and vaccination are generated, providing increasingly precise knowledge that can refine our estimates not only for currently available vaccines, but for vaccines of the future as well.

## Supplementary Material

Supplementary File

Supplementary File

Supplementary File

Supplementary File

Supplementary File

Supplementary File

Supplementary File

Supplementary File

## Data Availability

All data generated by this research has been made available from our Supplementary Materials or from the publicly accessible database Zenodo: https://doi.org/10.5281/zenodo.6027311 (75). Accordingly, all data from this research is accessible upon publication.
