# Supplementary material for: The durability of natural infection and vaccine-induced immunity against future infection by SARS-CoV-2
Source: Proc Natl Acad Sci U S A. 2022 Jul 15;119(31):e2204336119. doi: 10.1073/pnas.2204336119 (PMC9351502; doi:10.1073/pnas.2204336119)
Supplement: Supplementary File [file pnas.2204336119.sapp.pdf]

PNAS

Supplementary Information for

The durability of natural infection and vaccine-induced immunity against future infection by SARS-CoV-2

Jeffrey P. Townsend PhD*, Hayley B. Hassler MS, Pratha Sah PhD, Alison P. Galvani PhD, and Alex Dornburg PhD

*Corresponding author: Jeffrey P. Townsend
Email: Jeffrey.Townsend@Yale.edu

This PDF file includes:

Figures S1 to S2
Legends for Datasets S1 to S7

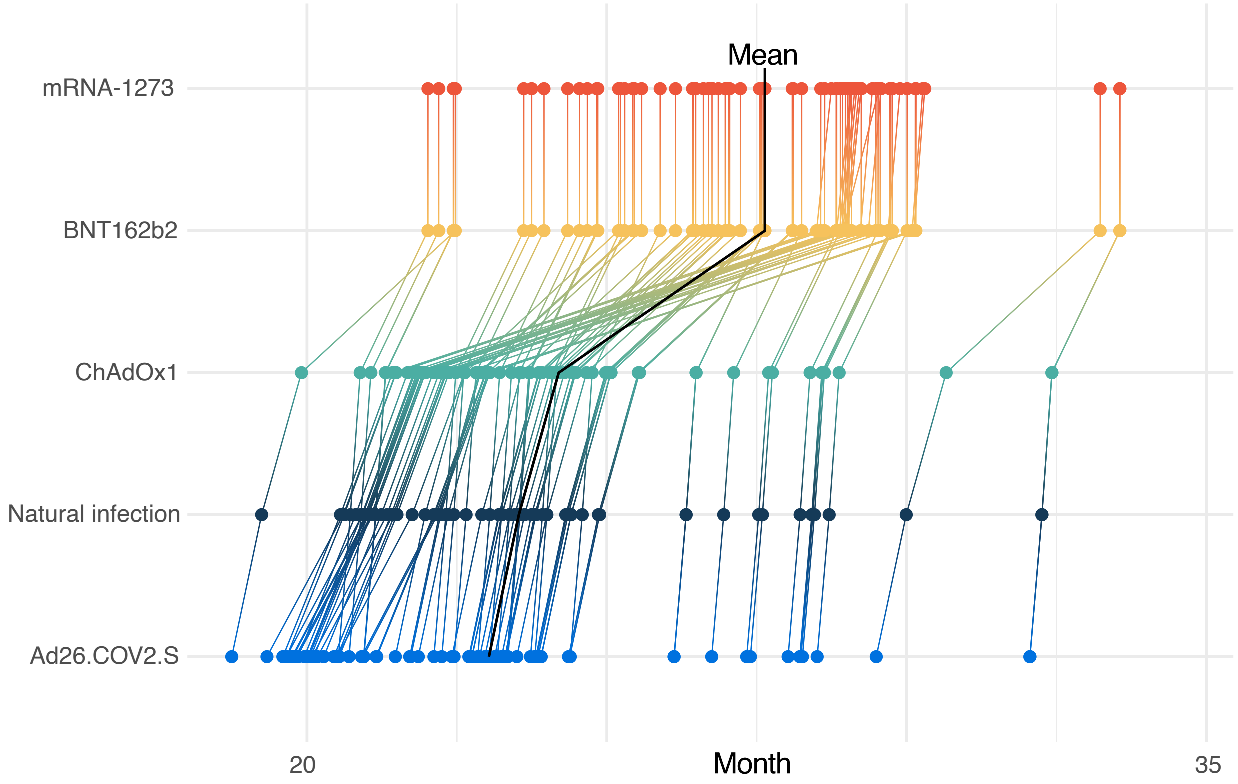

Fig. S1. Evaluation of the sensitivity of the median time to reinfection or breakthrough infection estimate based on the 84 different analysis combinations of alternate SARS-CoV-2 natural infection data (three datasets), alternate MERS-CoV natural infection data (two datasets), and alternate phylogenetic inferences (14 phylogenies).

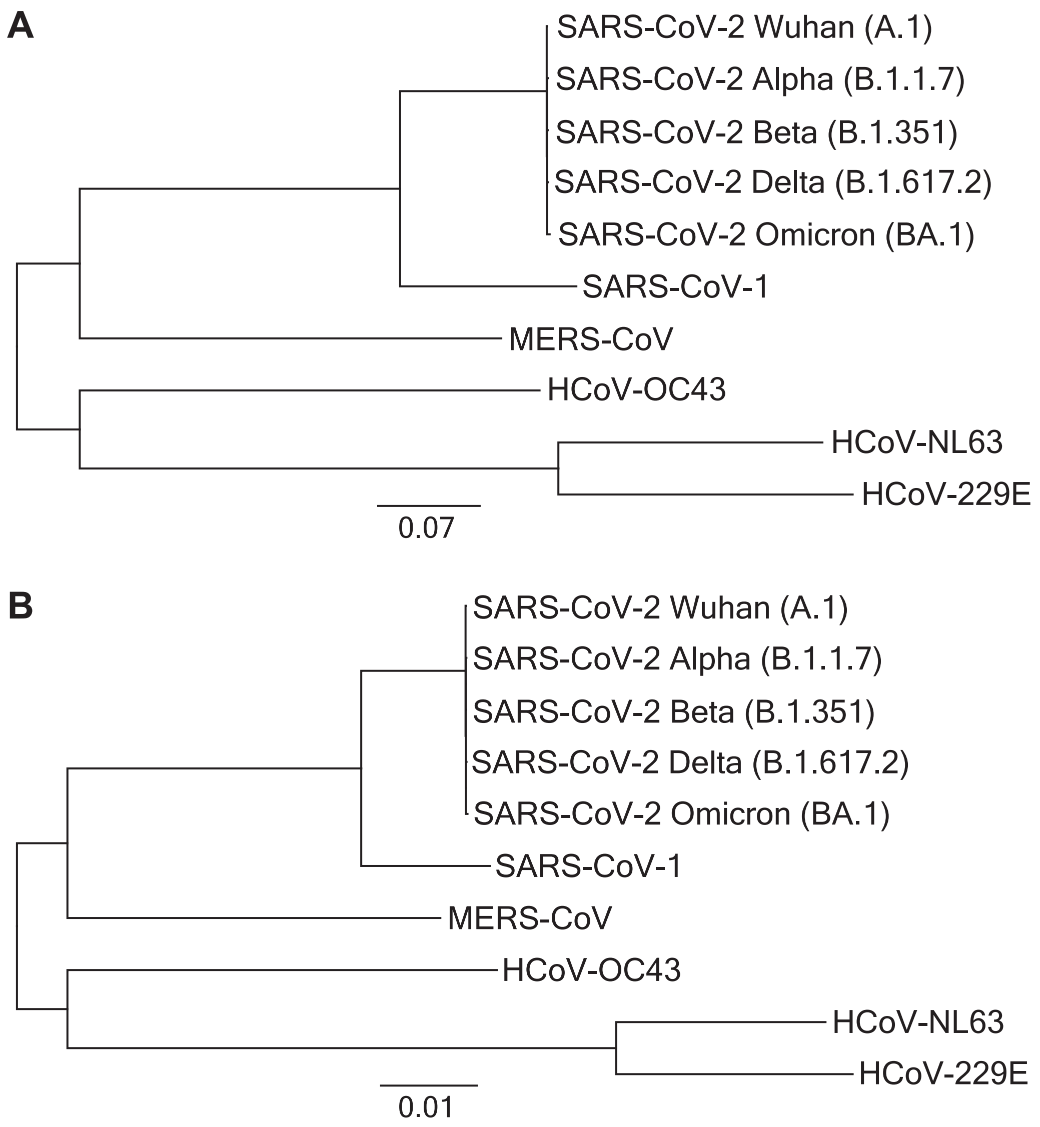

Fig. S2. (A) Molecular phylogeny and (B) phylogenetic chronogram produced by IQ-TREE on viral S, M, and ORF1b gene sequences. Bootstrap support was 100% for all nodes of these phylogenies.

Dataset S1 (separate file). Raw and average peak-normalized ELISA ODs for seasonal coronavirus antibodies.

Dataset S2 (separate file). Sample-size weighted mean antibody waning profile for BNT162b2.

Dataset S3 (separate file). Half-life to baseline estimates for natural infection.

Dataset S4 (separate file). Median time to reinfection/breakthrough infection estimates.

Dataset S5 (separate file). 5% quantile time to reinfection/breakthrough infection estimates.

Dataset S6 (separate file). 95% quantile time to reinfection/breakthrough infection estimates.

Dataset S7 (separate file). Assembly IDs for genome sequences used in study.
